# Hydrogen Sulfide and Polysulfides as Biological Mediators

**DOI:** 10.3390/molecules191016146

**Published:** 2014-10-09

**Authors:** Hideo Kimura

**Affiliations:** National Institute of Neuroscience, National Center of Neurology and Psychiatry, Kodaira, Tokyo 187-8502, Japan; E-Mail: kimura@ncnp.go.jp; Tel.: +81-42-346-1725; Fax: +81-42-346-1755

**Keywords:** H_2_S, H_2_S_n_, nitric oxide, CBS, CSE, 3MST, TRPA1, Nrf2, PTEN

## Abstract

Hydrogen sulfide (H_2_S) is recognized as a biological mediator with various roles such as neuromodulation, regulation of the vascular tone, cytoprotection, anti-inflammation, oxygen sensing, angiogenesis, and generation of mitochondrial energy. It is produced by cystathionine β-synthase (CBS), cystathionine γ-lyase (CSE), and 3-mercaptopyruvate sulfurtransferase (3MST). The activity of CBS is enhanced by *S*-adenosyl methionine (SAM) and glutathionylation, while it is inhibited by nitric oxide (NO) and carbon monoxide (CO). The activity of CSE and cysteine aminotransferase (CAT), which produces the 3MST substrate 3-mercaptopyruvate (3MP), is regulated by Ca^2+^. H_2_S is oxidized to thiosulfate in mitochondria through the sequential action of sulfide quinone oxidoreductase (SQR), sulfur dioxygenase, and rhodanese. The rates of the production and clearance of H_2_S determine its cellular concentration. Polysulfides (H_2_S_n_) have been found to occur in the brain and activate transient receptor potential ankyrin 1 (TRPA1) channels, facilitate the translocation of nuclear factor erythroid 2-related factor 2 (Nrf2) to the nucleus, and suppress the activity of phosphatase and tensin homolog (PTEN) by sulfurating (sulfhydrating) the target cysteine residues. A cross talk between H_2_S and NO also plays an important role in cardioprotection as well as regulation of the vascular tone. H_2_S, polysulfides, and their cross talk with NO may mediate various physiological and pathophysiological responses.

## 1. Introduction

Hydrogen sulfide (H_2_S) readily dissolves in water, and dissociates to H^+^, HS^−^, and S^2−^. Under physiological conditions, approximately 20% exist as H_2_S and the remaining 80% as HS^−^, with only trace amounts of S^2−^. The term “hydrogen sulfide” has been used to refer to H_2_S, HS^−^, and S^2−^ [1]. H_2_S was first detected in mammalian brains in 1989 [2,3,4]. The H_2_S concentrations reported in these studies were later found to strongly overestimate the true concentrations [5,6,7]. This discrepancy has been explained by the use of inappropriate methods to measure free H_2_S in the original studies. Nevertheless, the fact that the refined methods used in recent studies detected H_2_S, albeit at low concentrations, confirmed the existence of H_2_S in cells. A possible role for H_2_S as a neuromodulator in the brain, and its generation by cystathionine β-synthase (CBS), was demonstrated [1]. Subsequently, an additional function as a smooth muscle relaxant was reported for H_2_S, together with evidence that suggests it is also produced by cystathionine γ-lyase (CSE) [8,9]. Based on the observation that H_2_S is produced in the brains of CBS-knockout mice, 3-mercaptopyruvate sulfurtransferase (3MST) together with cysteine aminotransferase (CAT), which is identical to aspartate sulfurtransferase (AAT), was shown to produce H_2_S in the presence of thioredoxin [10,11,12]. Recently, we found a novel pathway consisting of 3MST and d-amino-acid oxidase (DAO) that generates H_2_S from d-cysteine [13].

Although the relaxation effect of H_2_S alone on the thoracic aorta was much weaker than that on the portal vein and ileum, H_2_S efficiently relaxes vascular smooth muscle in synergy with nitric oxide (NO) [8]. The chemical interaction of H_2_S and NO produces nitrosothiol, which releases NO in the presence of Cu^2+^ [14]. Nitrosoglutathione (GSNO), which also functions as a carrier of NO, releases NO in the presence of H_2_S to induce vascular smooth muscle relaxation [15]. Recently, it was demonstrated that H_2_S and nitrite interact with each other and produce HSNO and HNO [16]. A further reaction with H_2_S has been proposed to generate HSSNO, which releases NO and polysulfides to activate soluble guanylyl cyclase and relax vascular smooth muscle [17].

H_2_S also regulates the activity of NO synthetase (NOS) to control the production of NO. H_2_S facilitates the phosphorylation of the endothelial NOS (eNOS) activation site to increase NO production, whereas it does not induce the phosphorylation of its inhibitory site [18]. Through these effects, H_2_S protects cardiac muscle from ischemia/reperfusion injury. The mechanism is supported by the observations that NO availability is low in CSE-knockout mice, but can be rescued by H_2_S administration. In contrast, the activity of neuronal NOS (nNOS) is suppressed by H_2_S in colon smooth muscle [19]. In this tissue, endogenously produced NO is significantly greater in CSE-knockout mice than in the wild type.

The significance of polysulfides in H_2_S biology has recently been recognized; their role in the sulfuration (sulfhydration) of cysteine residues in target proteins to modify their activities has been documented [20,21]. Polysulfides were found to occur in the brain and to activate transient receptor potential ankyrin 1 (TRPA1) channels by sulfurating cysteine residues localized at the amino-terminus of the channels [22,23,24,25]. The regulation of phosphatase and tensin homolog (PTEN) activity by polysulfides was subsequently shown [26]. The transcription factor nuclear factor erythroid 2-related factor 2 (Nrf2) is normally sequestered in the cytosol by two molecules of Kelch-like ECH-associated protein 1 (Keap1). Once a reactive cysteine residue in Keap1 is sulfurated by polysulfides, Nrf2 is released by Keap1, after which it translocates to the nucleus, and upregulates the transcription of antioxidant genes [27]. Glutathione persulfide (GSSH) inhibits cytochrome *c* and scavenges hydrogen peroxide (H_2_O_2_) more efficiently than glutathione [28,29,30]. GSSH can be produced by the metabolism of H_2_S by sulfide quinone oxidoreductase (SQR) in mitochondria [31,32,33]. It may also be produced from cysteine persulfide (CysSSH), which has been proposed to be produced by CBS and CSE with cystine as a substrate, though the physiological relevance of the pathway has to be re-evaluated [30]. Polysulfides contain sulfane sulfur, which exists in a higher oxidation state than the sulfur atom in H_2_S and exerts various physiological effects.

## 2. The Regulation of Local H_2_S Concentrations

**Figure 1:**
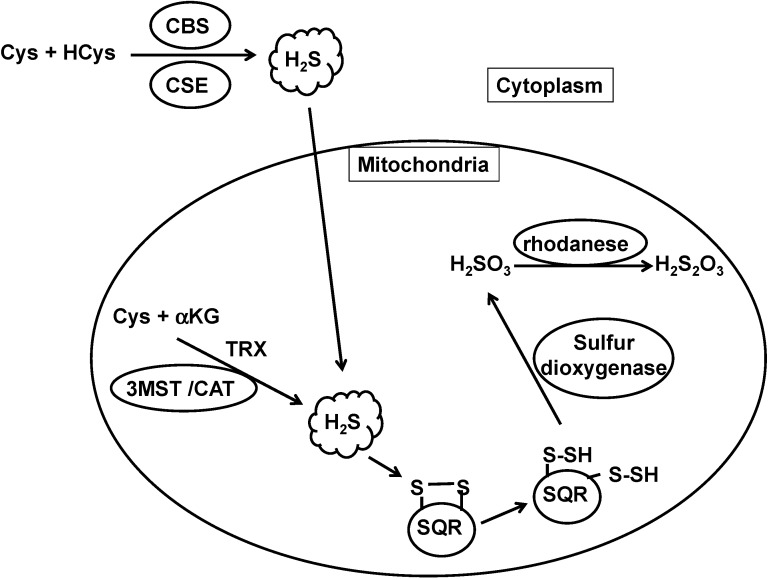
The metabolic turnover of H_2_S. H_2_S is produced by cystathionine β-synthase (CBS), cystathionine γ-lyase (CSE), and 3-mercaptopyruvate sulfurtransferase (3MST), and oxidized by sulfide quinone oxidoreductase (SQR) in mitochondria to produce persulfide. Sulfur dioxygenase oxidizes persulfide to sulfite (H_2_SO_3_), which is metabolized by rhodanese to produce thiosulfate (H_2_S_2_O_3_). The balance between H_2_S production and clearance of H_2_S determines its cellular concentration.

The concentration of H_2_S is determined by the balance between its production and clearance. H_2_S is produced by CBS, CSE, and 3MST and is metabolized by H_2_S oxidation pathways (Figure 1).

H_2_S production by CBS is enhanced by *S*-adenosyl methionine (SAM), which binds to the SAM binding site located at the carboxy-terminus [1,34]. The activity of CBS is also enhanced by glutathionylation of Cys346 [35]. Under oxidative stress, glutathione is consumed to protect cells, and Cys346 of CBS is oxidized to a sulfenic acid that then reacts with glutathione. Glutathionylated CBS increases the production of cysteine and H_2_S, which in turn promotes the production of glutathione. In contrast to the regulation by SAM and glutathionylation, the binding of NO and carbon monoxide (CO) to a heme group at the amino-terminus suppresses the activity of CBS [36]. This negative regulation plays an important role in the hypoxic brain [37], when the production of CO by heme oxygenase is decreased. The reduction of CO levels causes the de-suppression of CBS activity in astrocytes, which surround capillaries. CBS-derived H_2_S then relaxes the capillaries to recover blood flow and oxygen supply.

CSE was previously reported to be regulated by Ca^2+^/calmodulin [38]. However, this study was performed in the presence of 1–2 mM Ca^2+^, which corresponds to the extracellular Ca^2+^ concentration. Because CSE is a cytosolic enzyme, an investigation of the influence of intracellular Ca^2+^ concentrations on CSE activity was required. At steady state, the intracellular concentration of Ca^2+^ is approximately 100 nM; it is increased up to approximately 3 μM when cells are stimulated. In the presence of steady-state intracellular Ca^2+^ concentrations, CSE was found to be active, whereas it is inhibited by 50% in the presence of more than 300 nM Ca^2+^. It should be noted that CSE activity is suppressed by Ca^2+^ without the involvement of calmodulin [39].

The production of H_2_S via the 3MST/CAT pathway is also regulated by Ca^2+^ [40]. The activity of CAT is maximal at steady-state Ca^2+^ concentrations, whereas it is suppressed by Ca^2+^ in a concentration-dependent manner and is almost abolished when Ca^2+^ concentrations reach 3 μM. Calmodulin is not involved in the regulation of CAT. Because 3MST requires thioredoxin to produce H_2_S, the availability of thioredoxin and its redox turnover affect the H_2_S-producing activity of 3MST [11,12,41].

In contrast to the regulation of H_2_S-producing enzymes, that of H_2_S-metabolizing enzymes such as sulfide quinone oxidoreductase, persulfide dioxygenase, and rhodanese, is poorly understood.

H_2_S can also be released from bound sulfane sulfur. The time-course of this process as well as the amount of H_2_S released vary among tissues [6]. Homogenates of neurons and astrocytes release H_2_S in the presence of endogenous concentrations of cysteine and glutathione. However, the release of H_2_S from intact astrocytes, during neuronal excitation, has not yet been successfully detected.

## 3. Polysulfides as Biological Mediators

### 3.1. Effects of Polysulfides

Neurons are surrounded by astrocytes, which are glial cells that display neurotransmitter receptors. Presynaptic neurons release a neurotransmitter to postsynaptic neurons; some of them activate surrounding astrocytes, which in turn release gliotransmitters to modify synaptic activity. H_2_S induces Ca^2+^-influx in astrocytes by activating transient receptor potential (TRP) channels [42,43]. We subsequently found that polysulfide salts, *i.e.*, sodium tri- and tetrasulfide (Na_2_S_3_ and Na_2_S_4_), activate TRP channels in astrocytes much more potently than H_2_S, although the subtype of TRP channels was not identified [22]. TRPA1 channels were found to be activated by high concentrations of sodium hydrosulfide (NaHS) (1–10 mM) in sensory neurons of the urinary bladder and dorsal ganglion cells [24,44]. H_2_S is readily oxidized to polysulfides, and is sequentially oxidized to polysulfides with a varying number of sulfur atoms, until the number of sulfur atoms reaches eight; at that point, the sulfur molecules cyclize and separate from polysulfides (H_2_S_n_; see Equations (1) and (2)).

2nH_2_S + 1/2(2n−1)O_2_ = H_2_S_2n_ + (2n−1)H_2_O (1)

HS^−^ ↔ HSS^−^ ↔ HSSS^−^ ↔…..↔HS_7_^−^ → S_8_ (2)

It is possible that some of the H_2_S used in these studies was oxidized to H_2_S_n_, which in turn activated TRPA1 channels [21,23,45].

Using selective agonists and antagonists as well as siRNA targeting TRPA1 revealed that H_2_S_n_ activate TRPA1 channels in astrocytes much more potently than H_2_S [25]. The K_m_ value for the activation of TRPA1 channels in astrocytes is approximately 90 nM, and an HPLC analysis in the same study found approximately 20 μM polysulfides in the brain.

### 3.2. A Mechanism for Polysulfide Activity

Sulfuration modifies enzyme activity, which has been extensively studied between the 1960s and 1980s [46,47,48]. The process, in which H_2_S-derived sulfur attaches to reactive cysteine residues of target proteins, was named sulfhydration by Snyder and colleagues, while Toohey later suggested that the correct term is sulfuration [20,21]. Sulfuration of glyceraldehyde-3-phosphate dehydrogenase (GAPDH) by H_2_S increases its catalytic activity, and that of actin facilitates its polymerization. Consistently, dithiothreitol (DTT) treatment removes the added sulfur and diminishes the activity of these proteins [20]. ATP-dependent K^+^ channels are activated by sulfuration, and sulfuration of nuclear factor κB (NF-κB) facilitates its translocation to the nucleus. In contrast, protein tyrosine phosphatase 1B (PTP1B) is inhibited by sulfuration, thus regulating the endoplasmic reticulum stress response [49,50,51]. Parkin is an E3 ubiquitin ligase that is affected in Parkinson’s disease and causes accumulation of α-synuclein, a major component of Lewy bodies; Parkin is activated by sulfuration at Cys95 and Cys59 [52].

Zhu and colleagues proposed a role for H_2_S in the reduction of cysteine disulfide bonds rather than in the sulfuration of cysteine residues. H_2_S activates vascular endothelial growth factor receptor 2 (VEGFR2), which induces angiogenesis by reducing a disulfide bond between Cys1045 and Cys1024 [53]. Mass spectrometric analysis shows that H_2_S reduces the cysteine disulfide bond existing in the synthesized hexapeptide, but does not sulfurate any of the 20 free amino acids, including cysteine. The sulfuration of cysteine residues is only transiently observed as an intermediate during the H_2_S-mediated reduction of the disulfide bond, and the intermediate is immediately reduced to cysteine through an attack by a second HS^−^ molecule.

**Figure 2:**
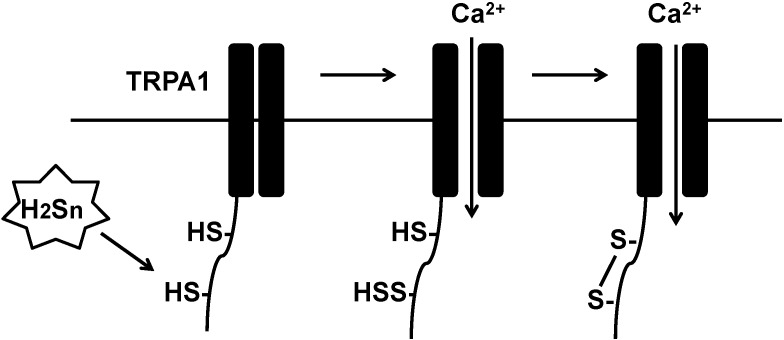
Polysulfides sulfurate cysteine residues of TRPA1 channels to modify their activity. Polysulfides sulfurate Cys422 and Cys622 at the amino-terminus of transient receptor potential ankyrin 1 (TRPA1) channels to activate them.

Atoms in the same oxidation state do not exchange electrons that results in no redox reaction. Because the oxidation state of sulfur in H_2_S and in free cysteine residues is −2, they do not react with each other. Ogawa *et al.* reported that the sites of TRPA1 channels sensitive to high concentrations of H_2_S are Cys422 and Cys622 at the amino-terminus, and that DTT abolishes the effect of H_2_S [24]. These observations suggest that Cys422 and Cys622 are sulfurated or bridged by a disulfide bond. H_2_S_3_ and H_2_S_4_ activate TRPA1 much more potently than H_2_S [25]. Because the oxidation state of sulfur in polysulfides such as H_2_S_3_ and H_2_S_4_ is 0, polysulfides can readily gain electrons from sulfur (−2) in cysteine residues. Therefore, both cysteine residues in TRPA1 are more likely to be sulfurated by polysulfides produced by H_2_S oxidation than by H_2_S per se. However, both cysteine residues may not be sulfurated simultaneously; in that case, the sulfurated cysteine residue may be attacked by the second free cysteine residue to produce the cysteine disulfide bond between Cys422 and Cys622 (Figure 2).

**Figure 3:**
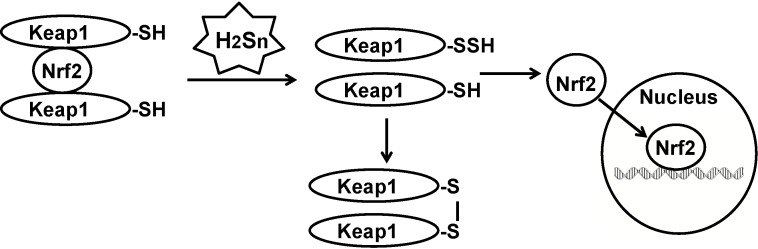
Sulfration of Keap1 by polysulfides release Nrf2 to the nucleus. Nuclear factor erythroid 2-related factor 2 (Nrf2) is sequestered in the cytosol by two molecules of Kelch-like ECH-associated protein 1 (Keap1). When Keap1 is sulfurated by polysulfides, Nrf2 is released and translocates into the nucleus, where it upregulates the transcription of antioxidant genes.

There are other examples for polysulfide-mediated sulfuration that were previously ascribed to H_2_S. Lefer and colleagues initially reported that H_2_S facilitates the translocation of Nrf2 to the nucleus to upregulate the transcription of antioxidant genes [54]. Wang and colleagues showed that H_2_S sulfurates Keap1 to release Nrf2 to the nucleus [55]. We showed that polysulfides sulfurate Keap1 (Figure 3) [27]. Polysulfides, but not H_2_S, inhibit the activity of lipid phosphatase and tensin homolog (PTEN) by inducing the formation of a cysteine disulfide bond [26]. In the latter study, mass spectrometric analysis revealed only the cysteine disulfide bond, but not the trisulfide bond suggested by Toohey [21].

### 3.3. Glutathione Polysulfide

Massey *et al.* initially reported that glutathione persulfide (GSSH or GSS^−^) produced from glutathione trisulfide (GSSSG) reduces cytochrome *c* more efficiently than glutathione (GSH), and that cysteine trisulfide has a similar effect [28]. The effect of GSSH on cytochrome *c* was confirmed, and a similar reductive effect was observed on papain [29]. GSSH produced from GSSSG scavenges H_2_O_2_ more efficiently than GSH [30].

GSH receives a sulfur atom from H_2_S through the action of SQR to form GSSH in mitochondria [31,32,33]. An alternative pathway to produce cysteine persulfide (CysSSH) and GSSH has been proposed [30]. CBS and CSE metabolize cystine to CysSSH, which produces GSSH via the exchange reaction with GSH. However, the physiological relevance of this pathway needs to be re-evaluated. CSE has a high affinity for cystine, the extracellular form of cysteine, with a K_m_ value of 30–70 μM. However, CBS and CSE localize to the cytosol, which contains concentrations of cystine that are insufficient to allow the enzymatic reaction [56]. Less than 0.2 μM cystine exists in the cytosol, with a few exceptions such as the A549 cell line, which contains approximately 12 μM cystine in the cytosol [30]. Even the extracellular concentration of cystine is only approximately 40 μM in human blood [57]. The *in vitro* generation of CysSSH by CSE and CBS was examined in the presence of millimolar concentrations of cystine, which greatly exceed physiological concentrations [30].

## 4. Cross Talk of H_2_S and NO

H_2_S relaxes vascular smooth muscle in synergy with NO, and a similar synergistic effect is observed in ileum [8,58]. NO also enhances the production of H_2_S and upregulates the transcription of CSE [9]. Lefer and colleagues recently showed that H_2_S activates eNOS by inducing the phosphorylation of its activation site; the subsequent increase in the production of NO protects the heart from ischemia/reperfusion injury [18]). This mechanism was confirmed by the observation that the administration of H_2_S did not confer cardioprotection in eNOS-deficient mice. In contrast, nNOS is inhibited by H_2_S in colon smooth muscle [19], as indicated by the observation that the endogenous generation of NO is significantly decreased in wild-type mice compared with CSE-knockout mice.

The chemical interaction between H_2_S and NO produces several potential intermediates. Nitrosothiol releases NO in the presence of Cu^2+^. GSNO, which is an intermediate or a carrier of NO, releases NO in the presence of H_2_S [14,15]. H_2_S and nitrite, in the presence of human umbilical vein endothelial cells or Fe^3+^-porphyrins, produce the intermediate HSNO, which in turn generates either NO and the HS radical, or HNO by a further reaction with H_2_S [16]. Feelisch and colleagues reported that HSNO may not effectively release NO because the S-N bond is too strong. They demonstrated that nitrosopersulfide (SSNO^−^), which is more stable than HSNO, efficiently releases NO and polysulfides to activate soluble guanylyl cyclase and relax smooth muscle [17]. These studies on the chemical interaction of H_2_S and NO were performed in the presence of millimolar concentrations of NaHS, which is why their physiological relevance needs to be further examined.

## 5. Conclusions

The role of H_2_S as a physiological mediator has been extensively studied in various tissues and organs. Although the steady-state concentrations of H_2_S have been re-evaluated and found to be much lower than those initially reported, neither the range of concentration changes nor the physiological stimuli to induce such changes have been understood. The balance between the production and clearance of H_2_S determines its concentration [59,60]. *S*-adenosyl methionine and glutathionylation enhance the activity of CBS, whereas NO and CO suppress it. CSE and CAT are regulated by Ca^2+^. In contrast, the regulation of H_2_S-degrading enzymes such as SQR and sulfur dioxygenase is only poorly understood.

Polysulfides have recently been recognized as potential physiological mediators. They have been found to occur in tissues and to activate channels, enzymes, and transcription factors through the mechanism of sulfuration (sulfhydration). However, a number of issues remain to be clarified, such as the production and degradation pathways of polysulfides and their regulatory mechanisms, as well as potential physiological stimuli that induce those regulatory mechanisms.

The cross talk between H_2_S and NO has also been extensively studied. A synergistic effect of H_2_S and NO was initially found. Recently, it was demonstrated that H_2_S acts as a stimulator of the release of NO, which is the final effecter molecule to the target proteins. In addition, the reaction of H_2_S with NO, which gives rise to highly reactive substances, such as HSNO, GSNO, HNO, and HSSNO has been proposed. The production of the latter substances will have to be re-evaluated under physiological conditions.

A better understanding of the regulation of their production as well as their mechanisms of action will help unveil the physiological roles of H_2_S and related molecules.
